# Supplementary figures and images for: Phosphoproteomic mapping reveals distinct signaling actions and activation of muscle protein synthesis by Isthmin-1
Source: eLife. 2022 Sep 28;11:e80014. doi: 10.7554/eLife.80014 (PMC9592085; doi:10.7554/eLife.80014)

Fig 1B

S473  
p-AKT

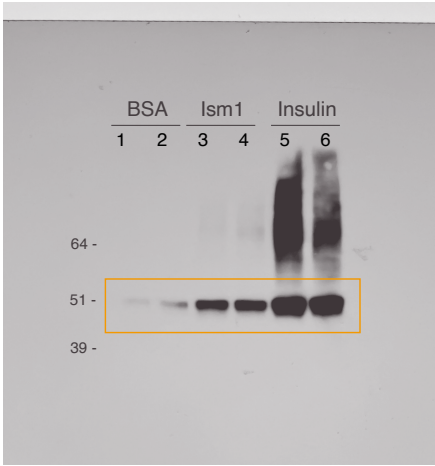

AKT

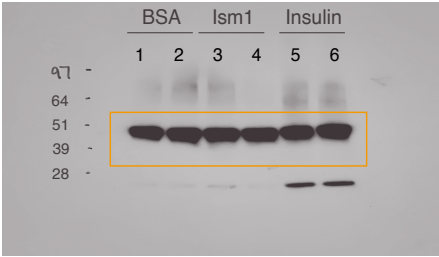

Loaded an extra  
lane for sample 6

$\beta$ -actin

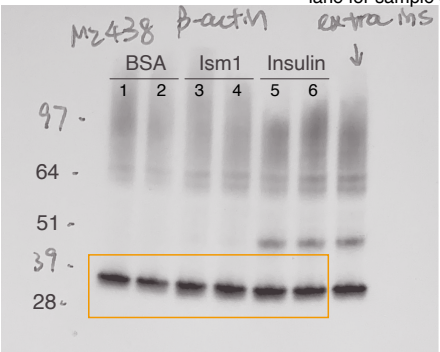

Supplement: Figure 1—source data 3. [file elife-80014-fig1-data3.pdf]

**Fig 3A**

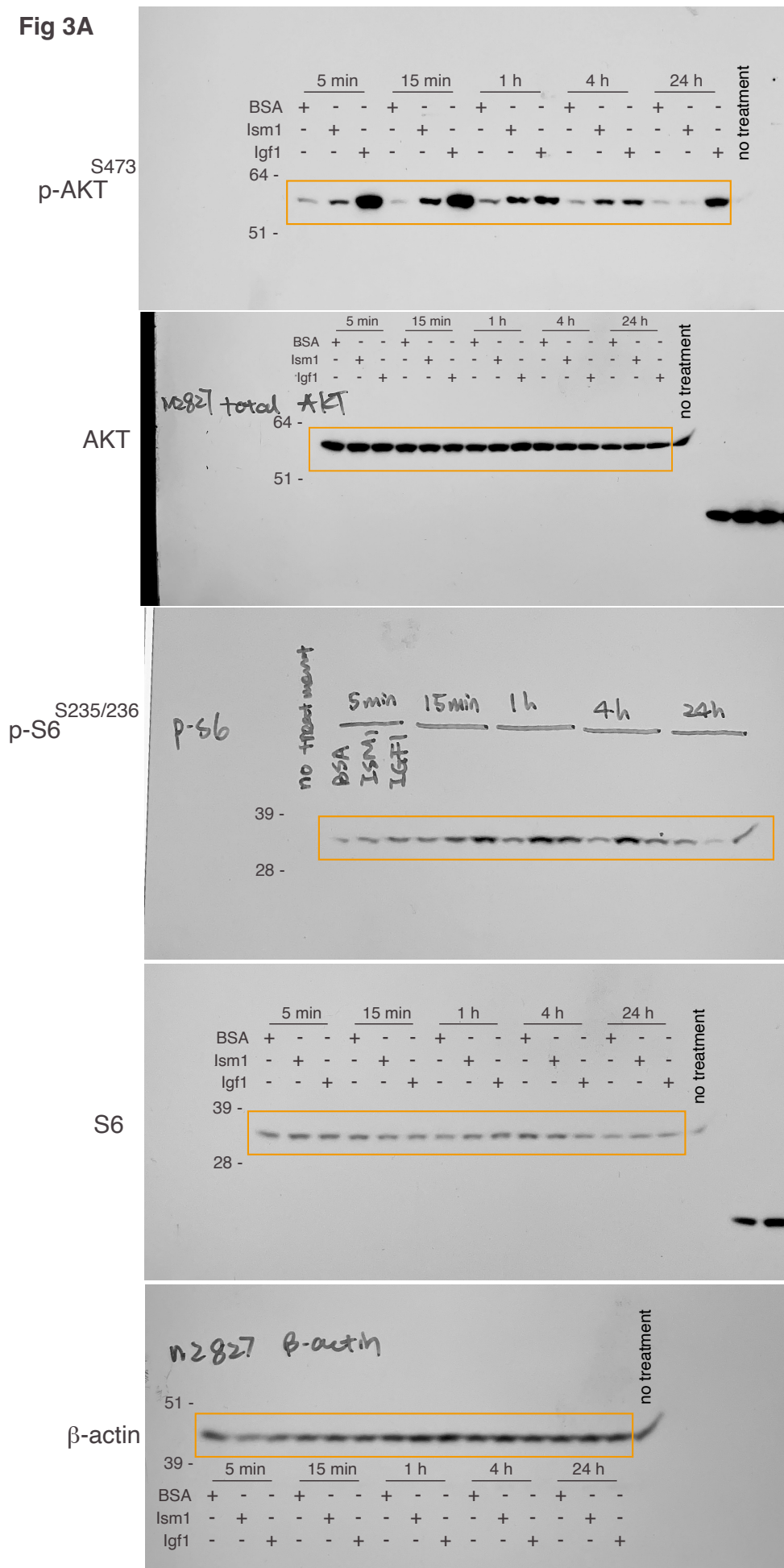

Fig 3C

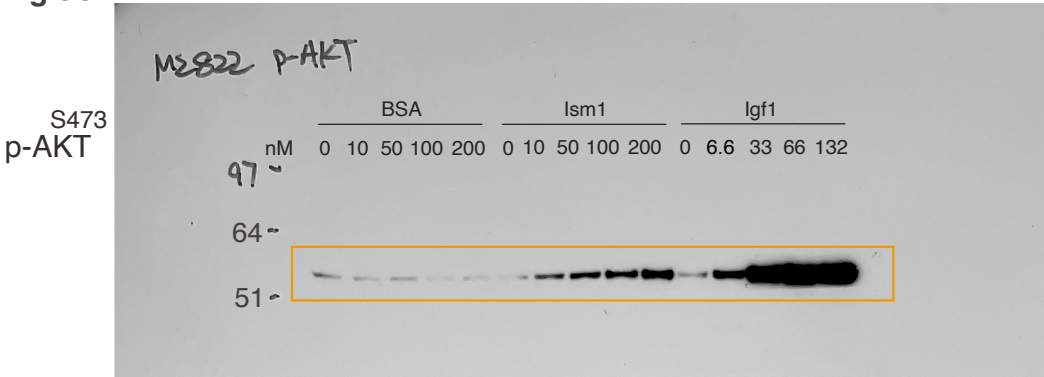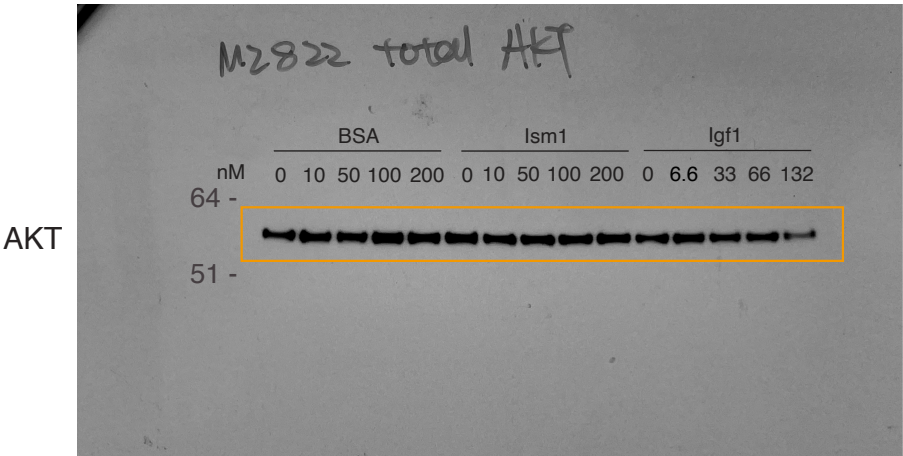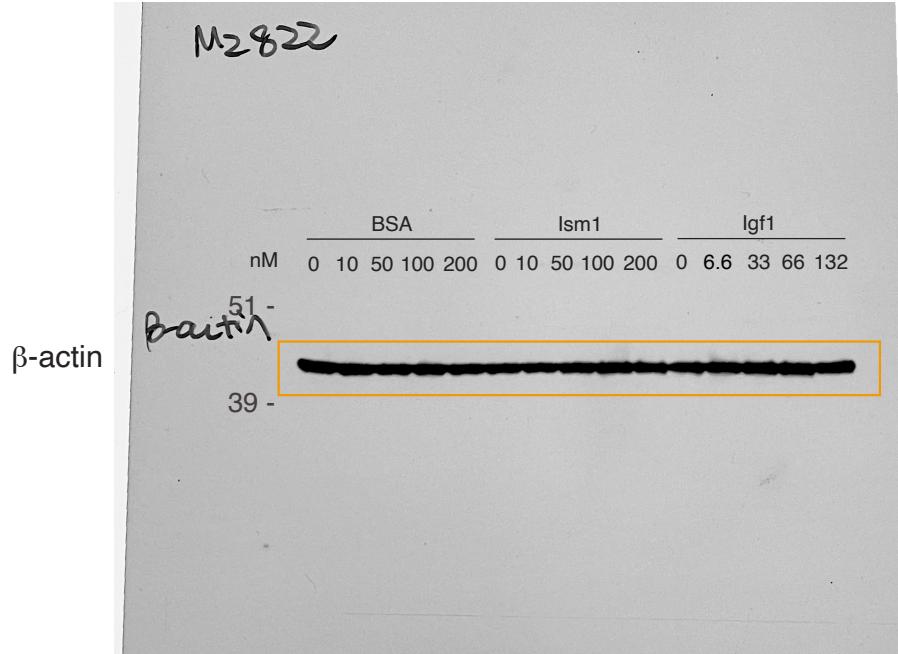

Supplement: Figure 3—source data 2. [file elife-80014-fig3-data2.pdf]

Fig 5E

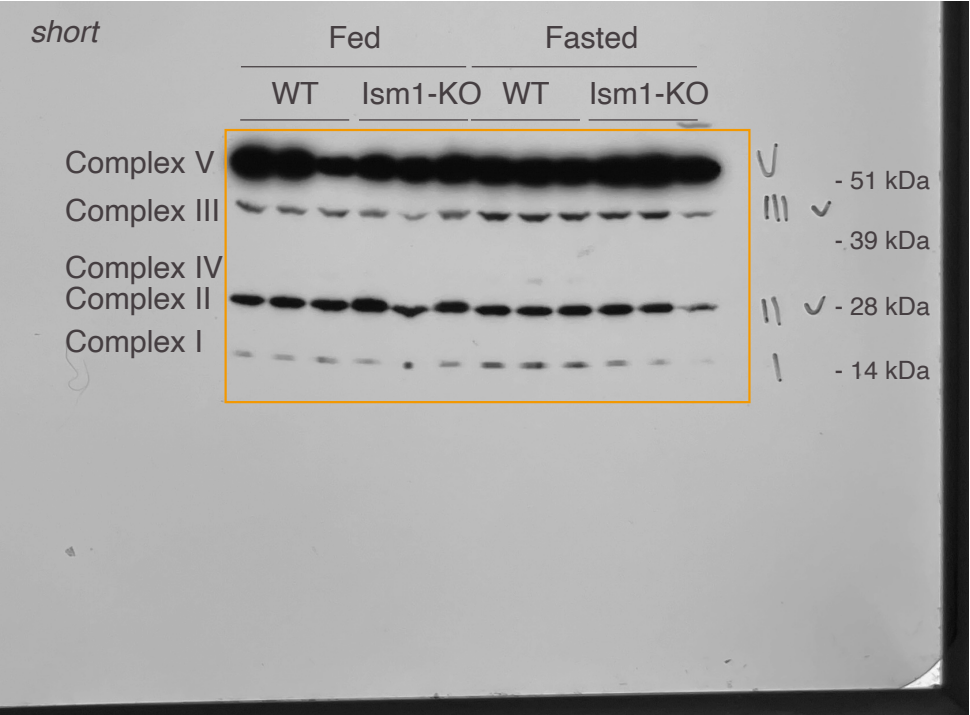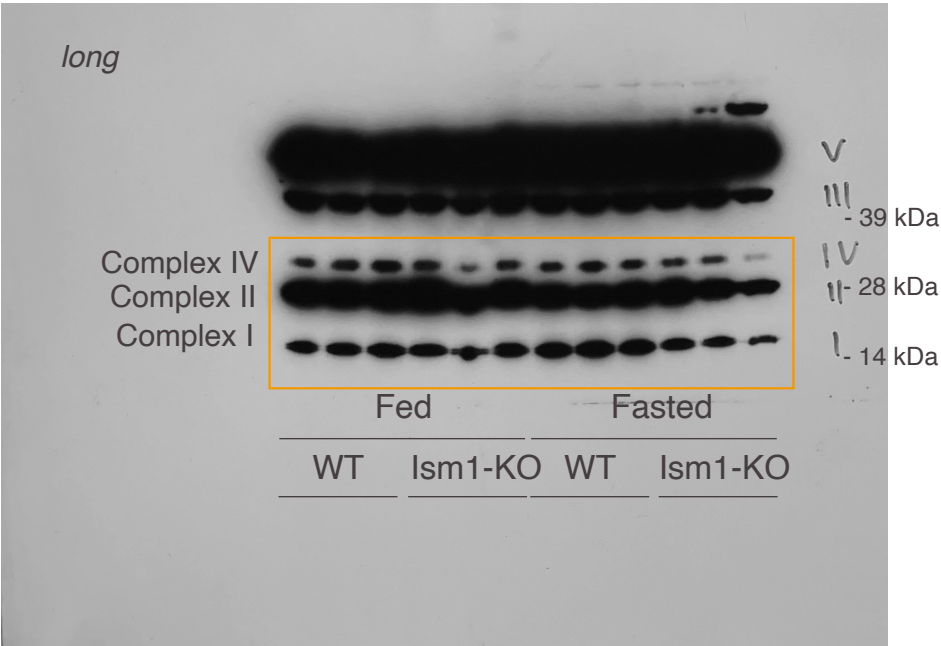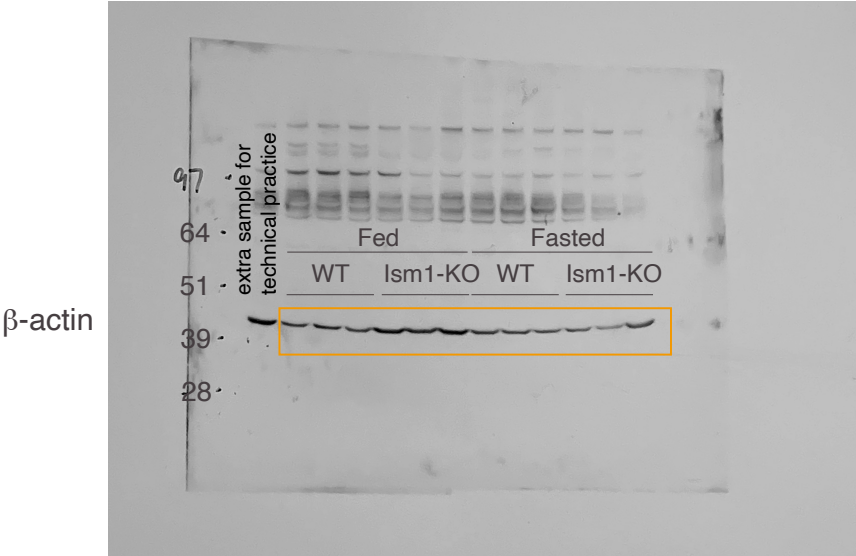

Supplement: Figure 5—source data 2. [file elife-80014-fig5-data2.pdf]

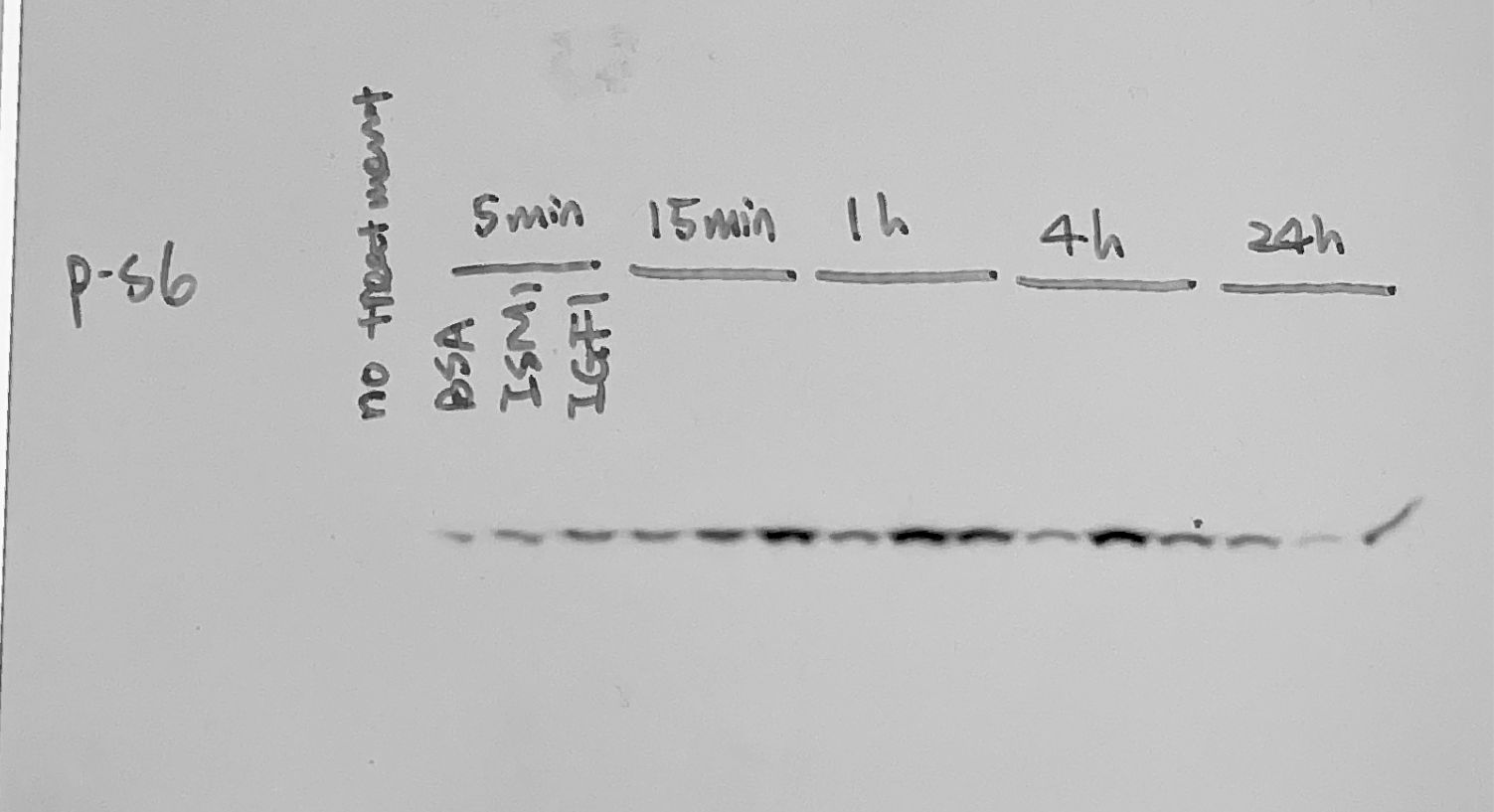

Supplement: Source data 1. [file elife-80014-data1.zip › Source data 1_western images/Figure 3A_pS6.png]

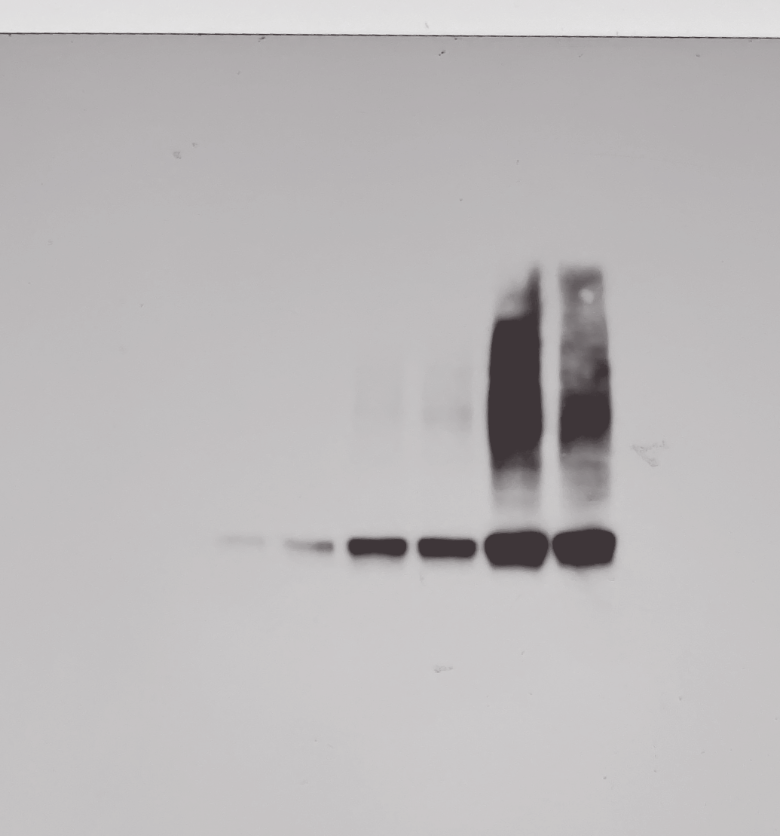

Supplement: Source data 1. [file elife-80014-data1.zip › Source data 1_western images/Figure 1B_pAKT.png]

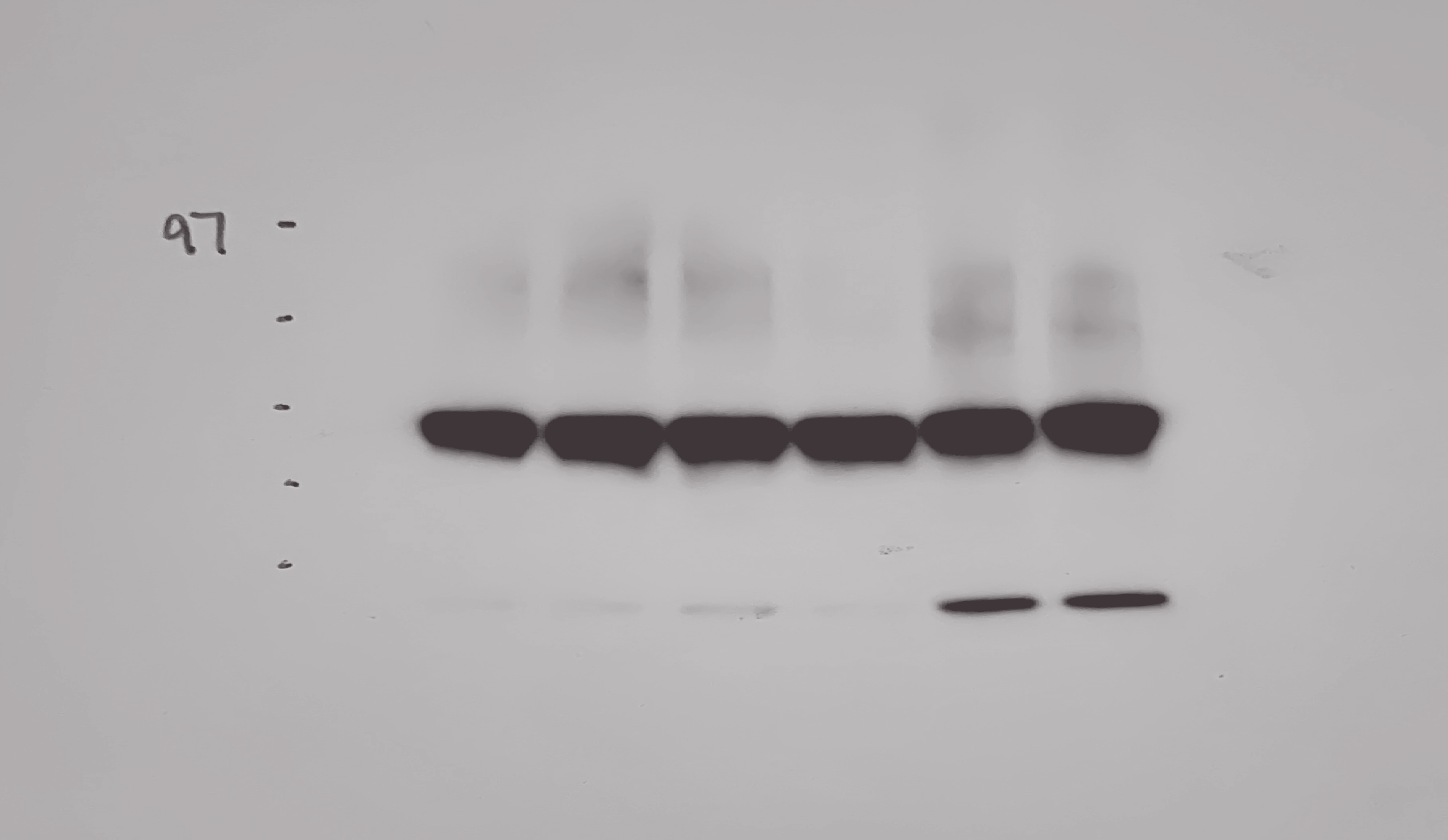

Supplement: Source data 1. [file elife-80014-data1.zip › Source data 1_western images/Figure 1B_AKT.png]

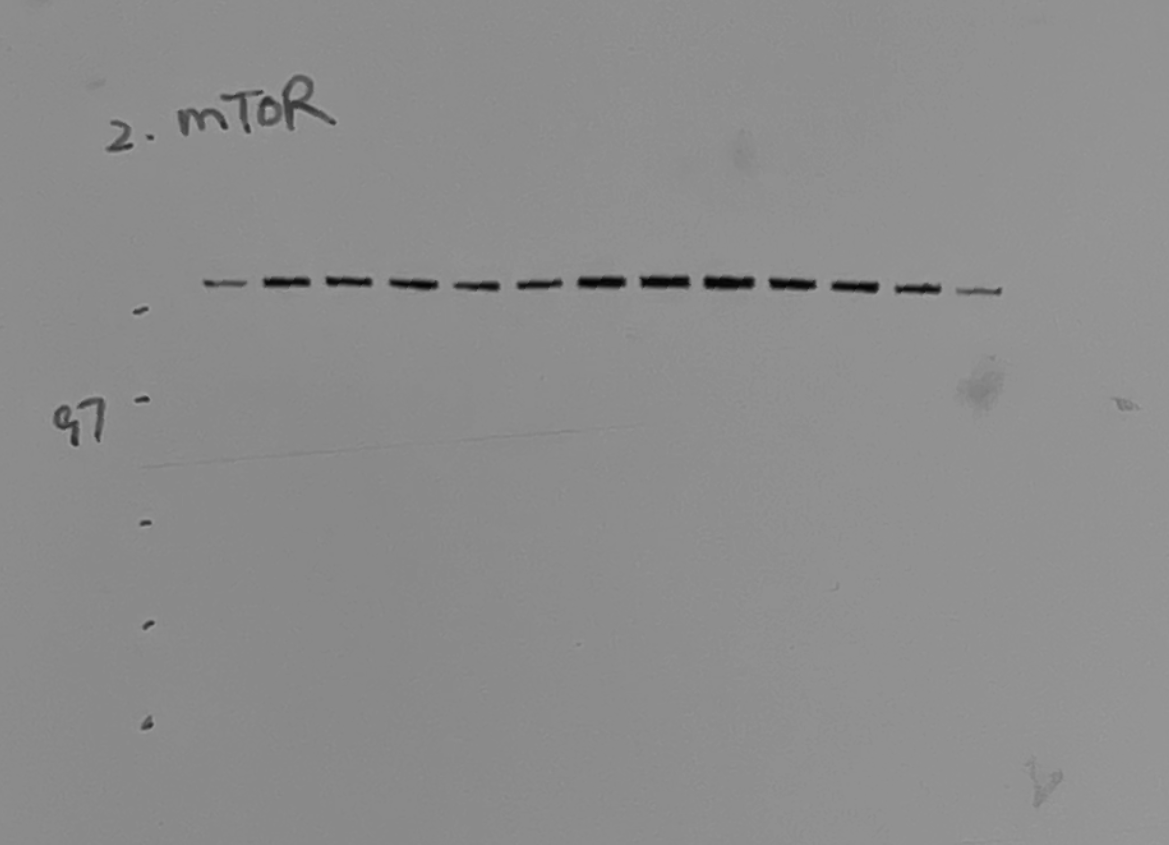

Supplement: Source data 1. [file elife-80014-data1.zip › Source data 1_western images/Figure 6G_mTOR.png]

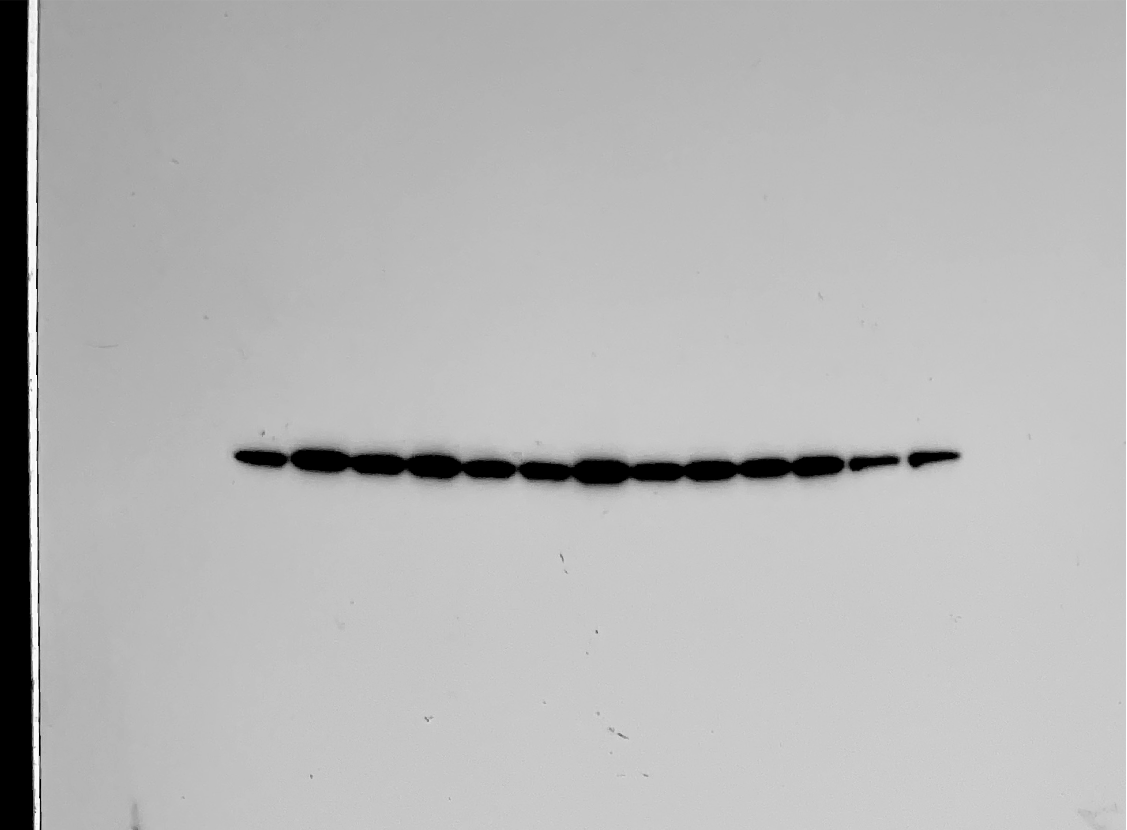

Supplement: Source data 1. [file elife-80014-data1.zip › Source data 1_western images/Figure 6G_S6.png]

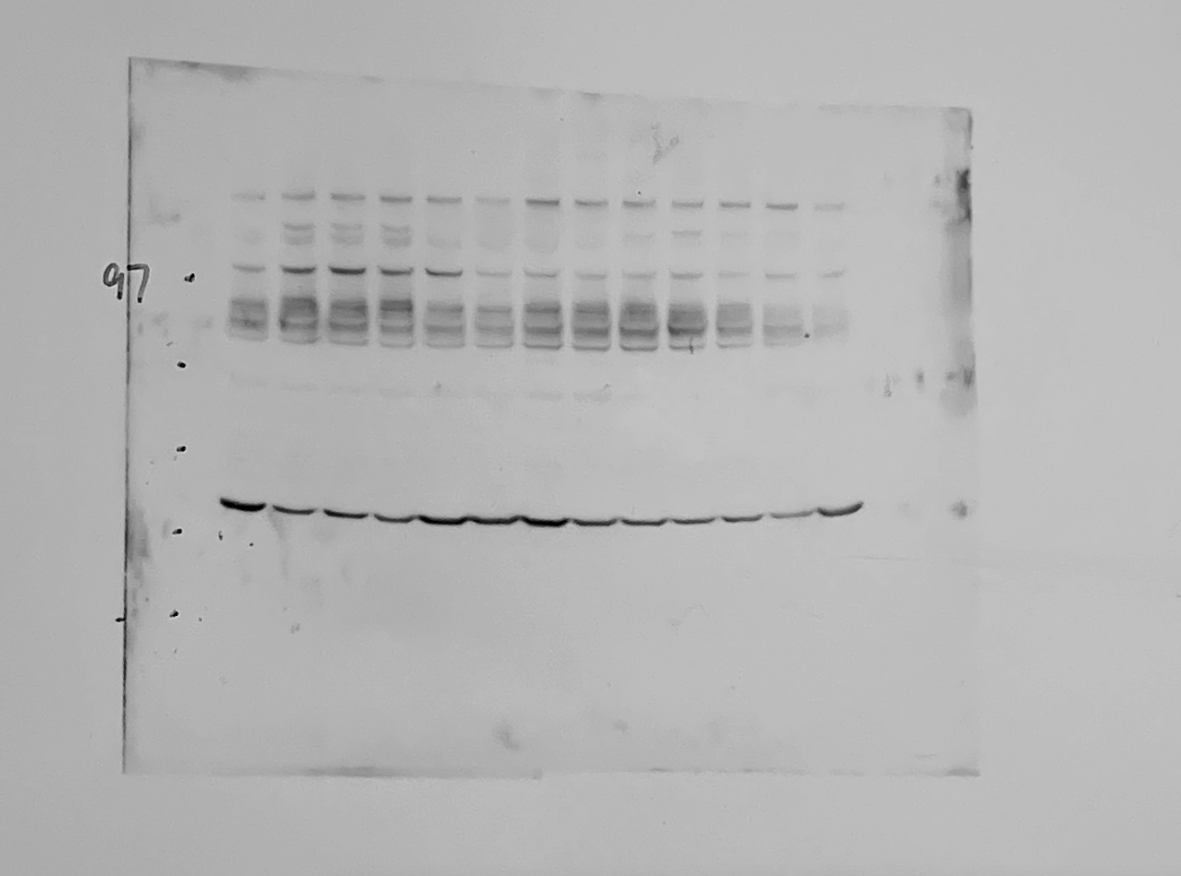

Supplement: Source data 1. [file elife-80014-data1.zip › Source data 1_western images/Figure 5E_actin.png]

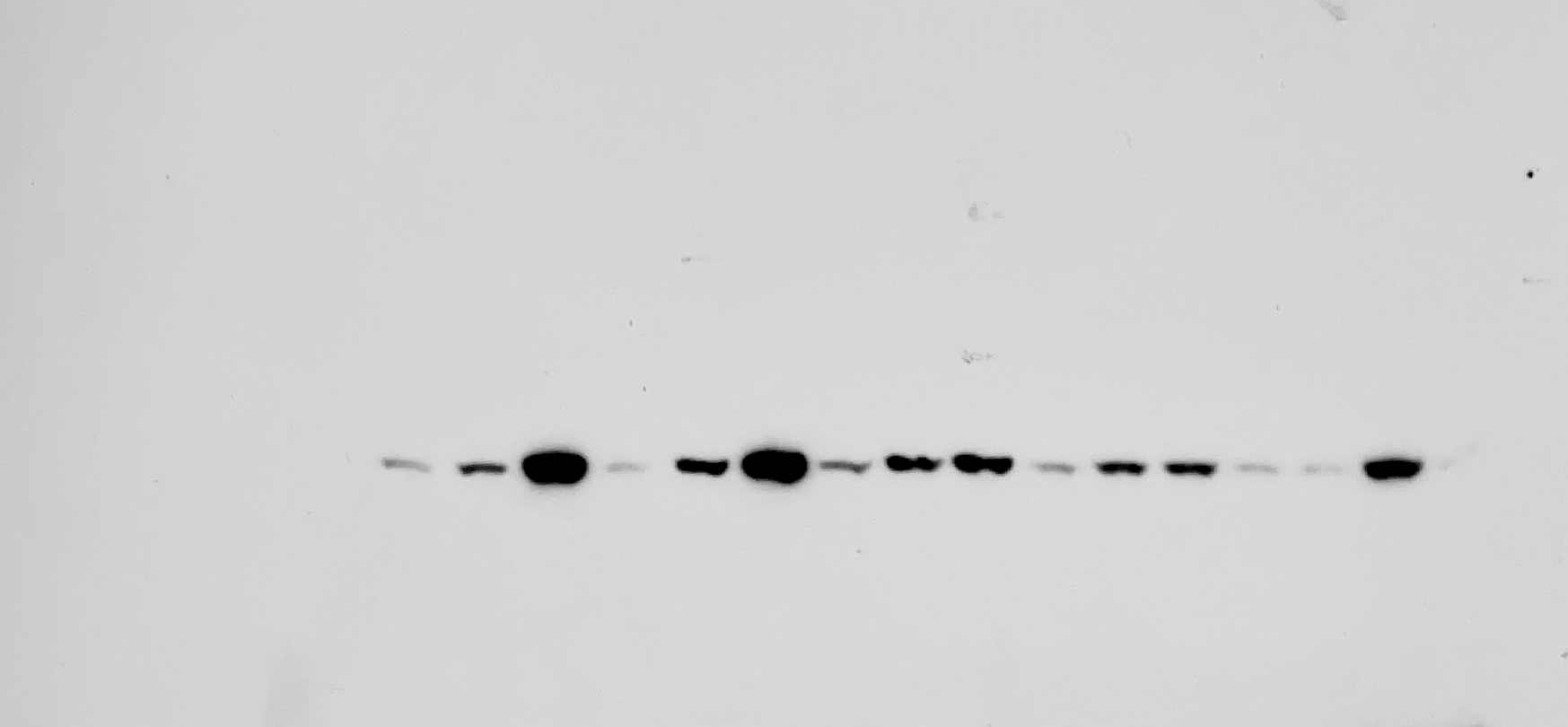

Supplement: Source data 1. [file elife-80014-data1.zip › Source data 1_western images/Figure 3A_pAKT.png]

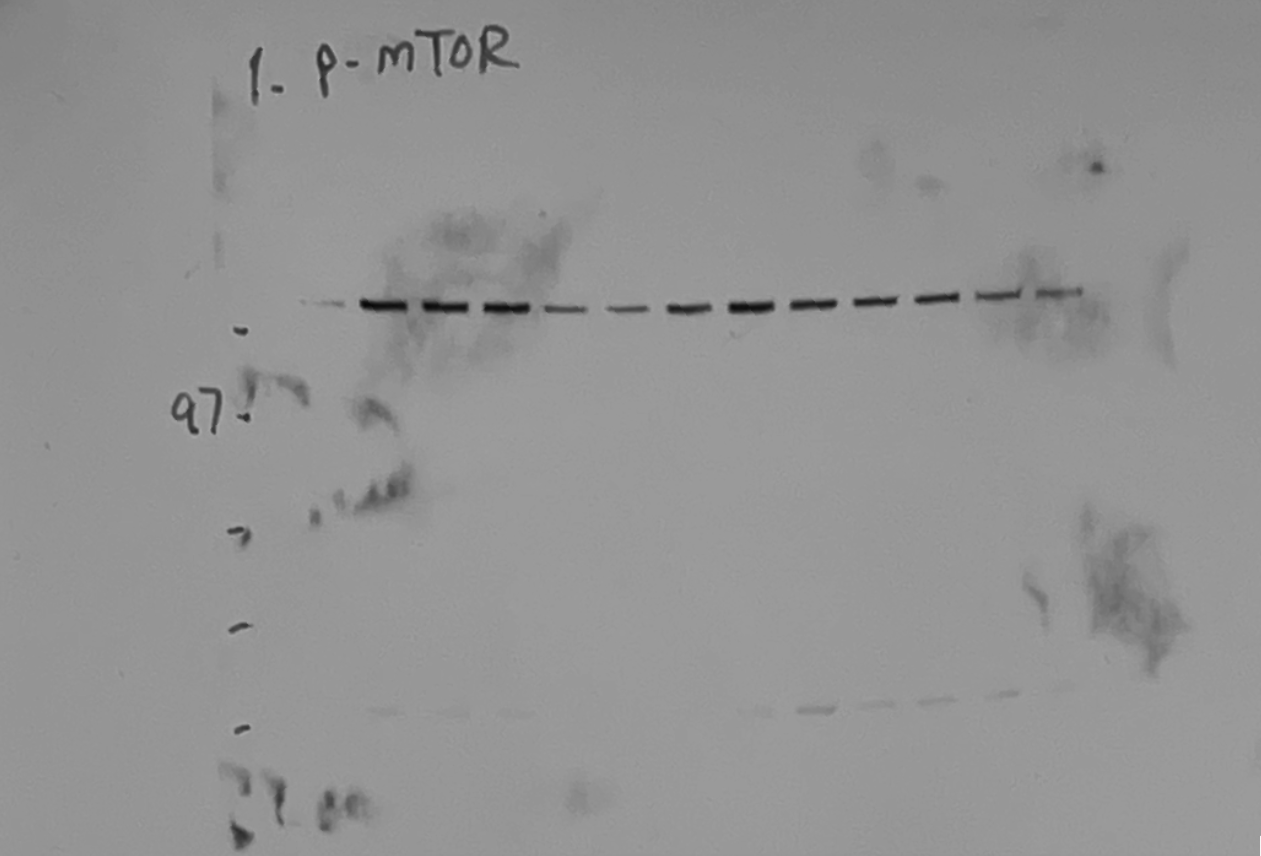

Supplement: Source data 1. [file elife-80014-data1.zip › Source data 1_western images/Figure 6G_pmTOR.png]

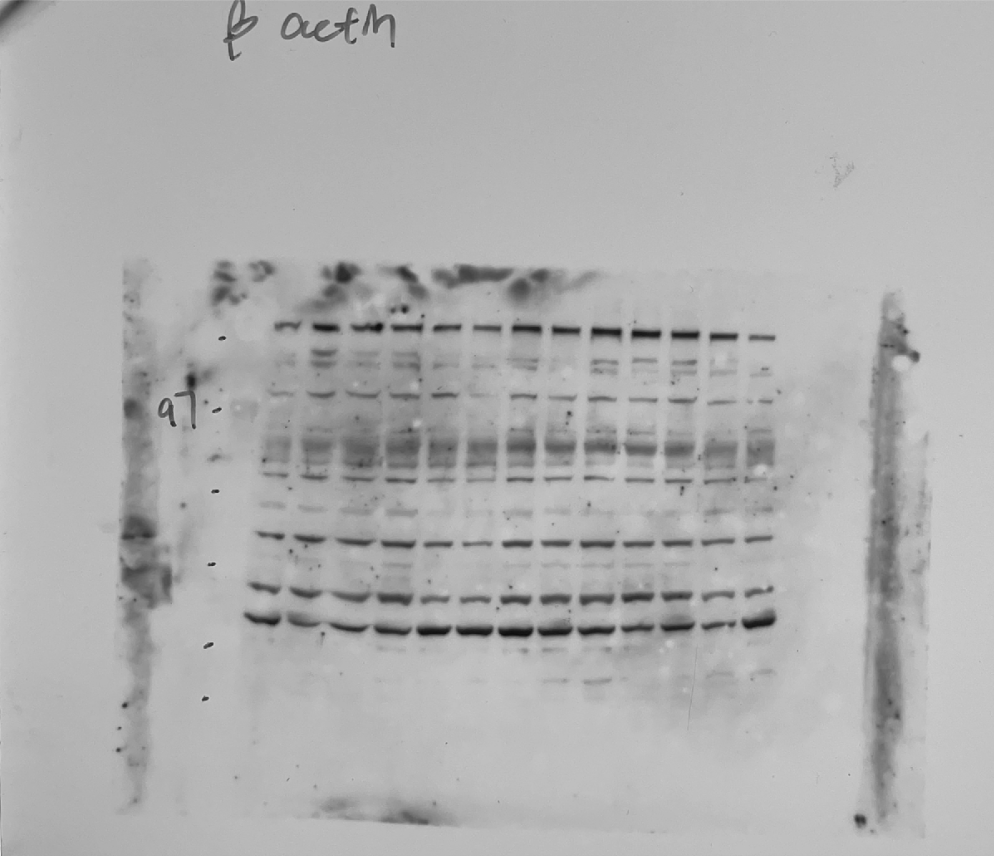

Supplement: Source data 1. [file elife-80014-data1.zip › Source data 1_western images/Figure 6G_actin.png]

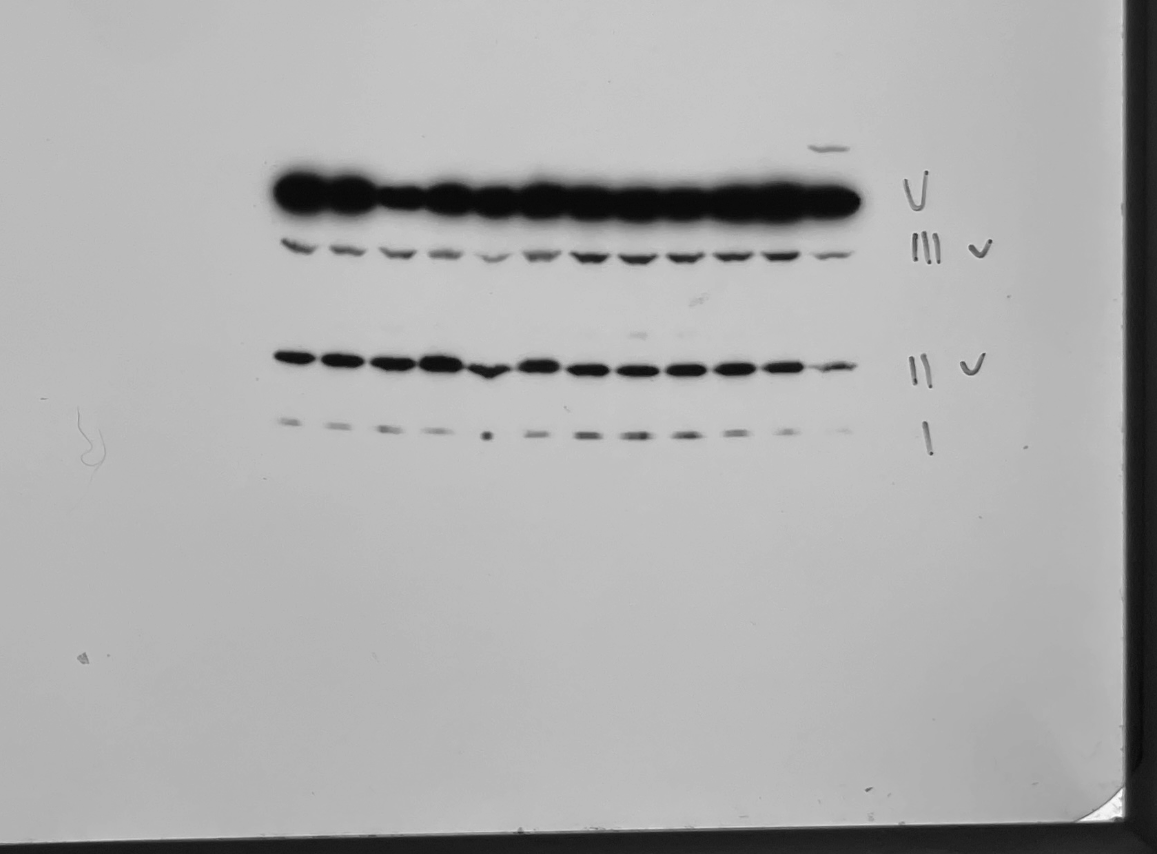

Supplement: Source data 1. [file elife-80014-data1.zip › Source data 1_western images/Figure 5E_Complex_short.png]

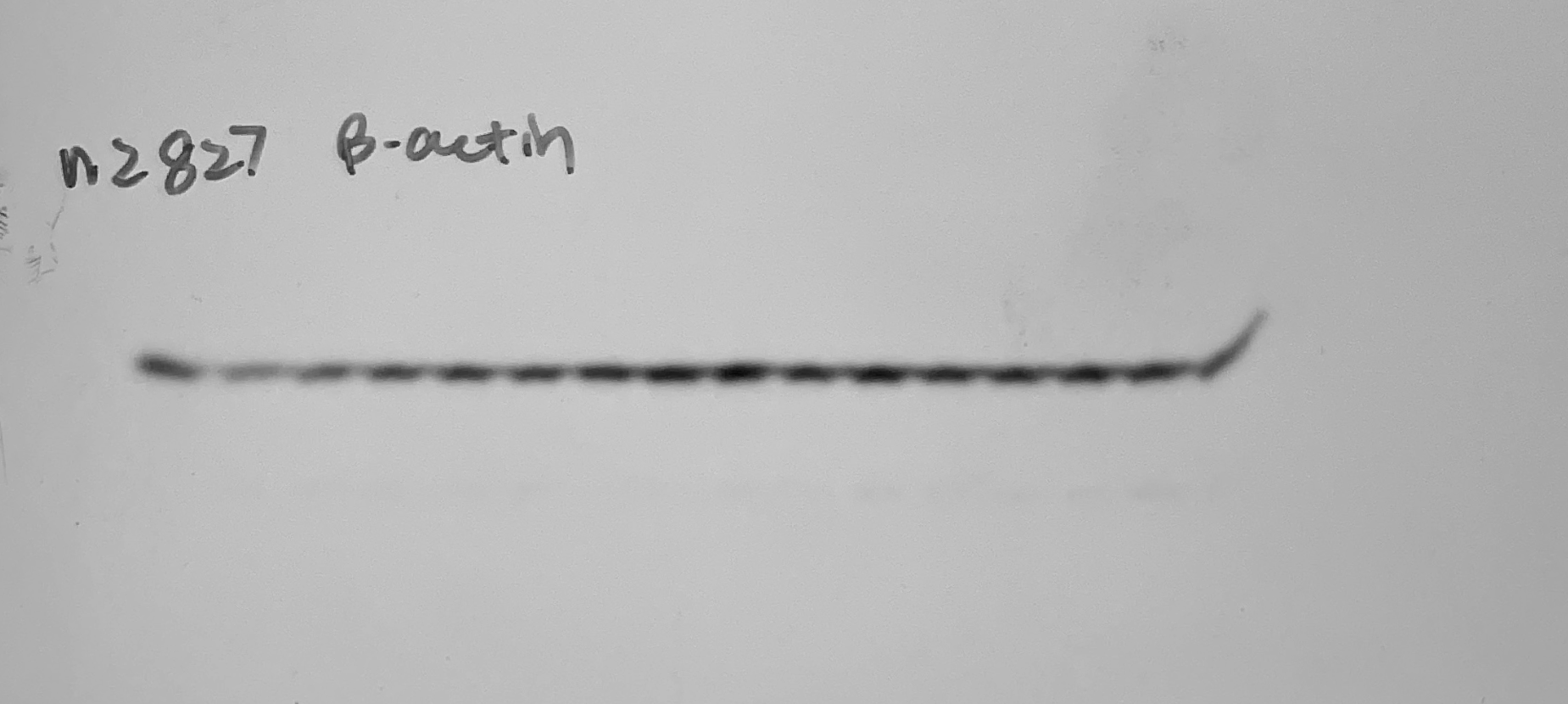

Supplement: Source data 1. [file elife-80014-data1.zip › Source data 1_western images/Figure 3A_actin.png]

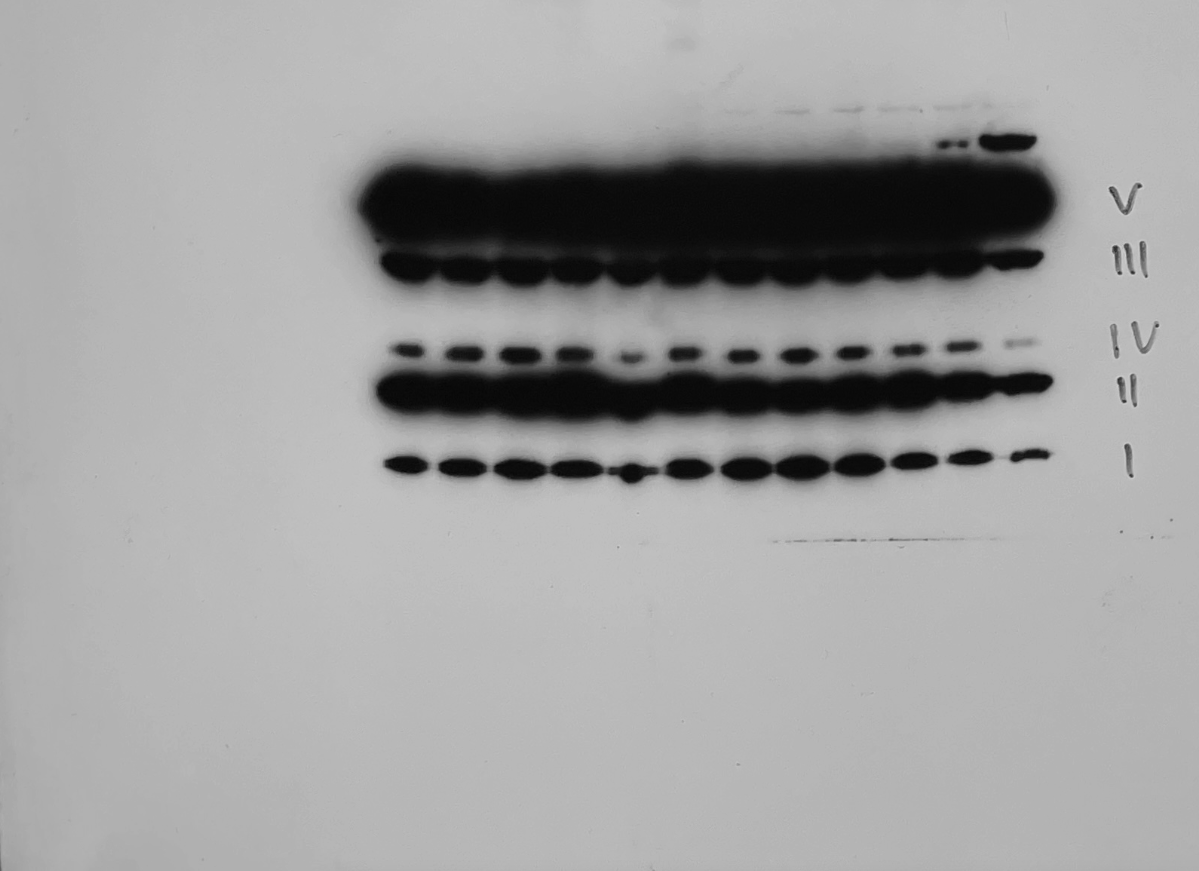

Supplement: Source data 1. [file elife-80014-data1.zip › Source data 1_western images/Figure 5E_Complex_long.png]

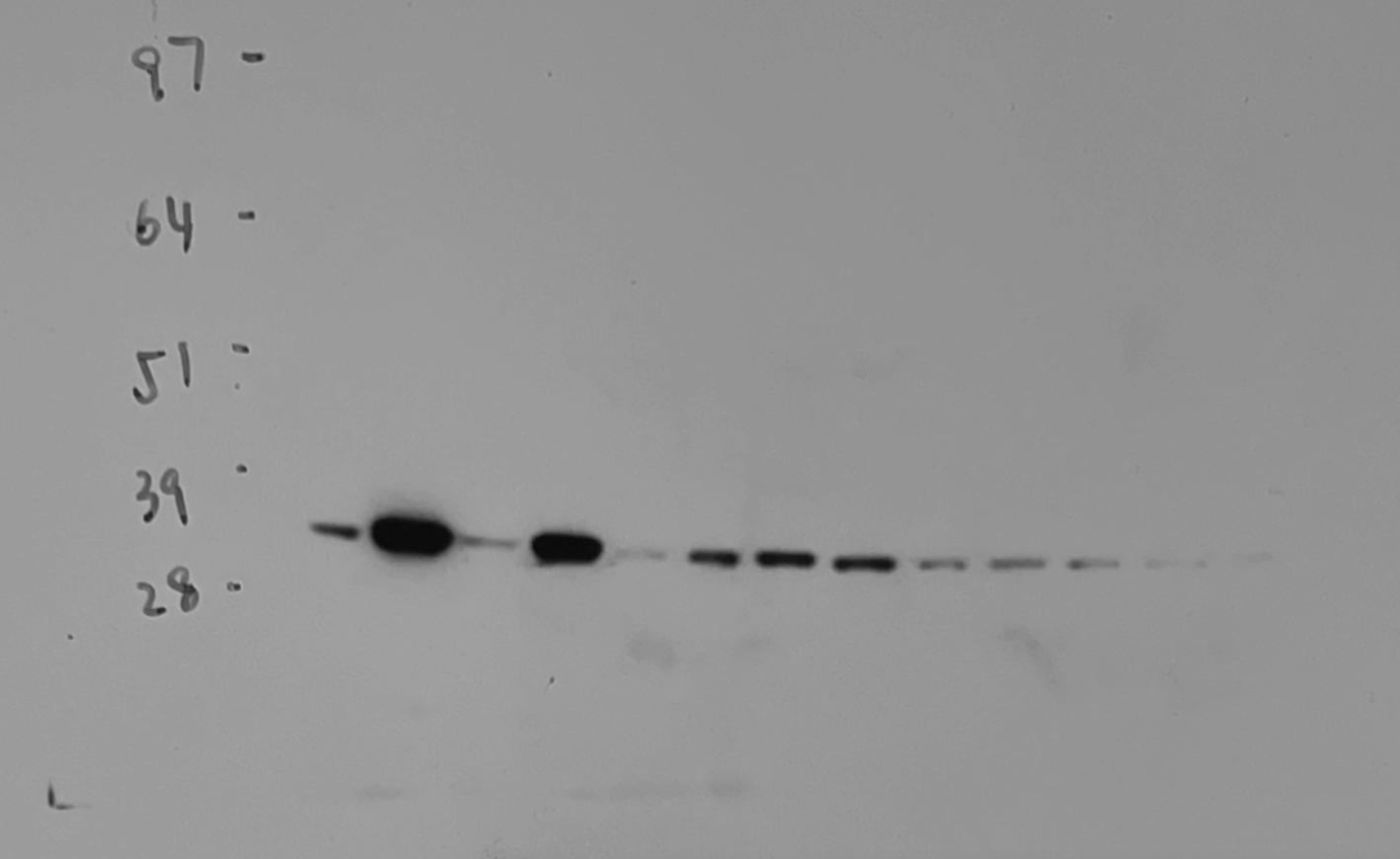

Supplement: Source data 1. [file elife-80014-data1.zip › Source data 1_western images/Figure 6G_pS6.png]

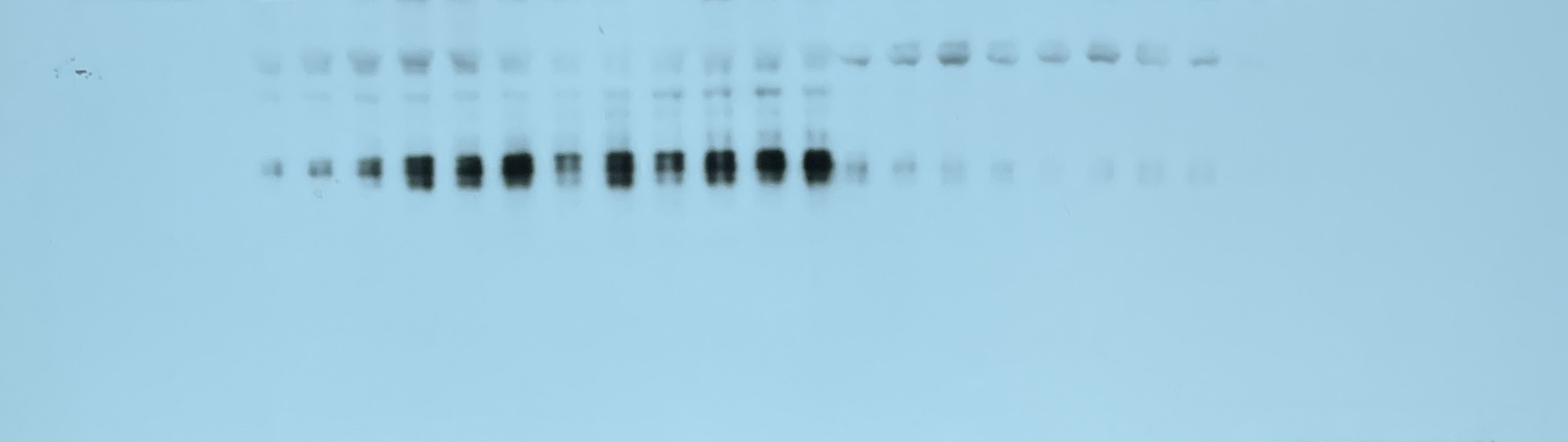

Supplement: Source data 1. [file elife-80014-data1.zip › Source data 1_western images/Figure 6F_p27_revision_Sep2.jpg]

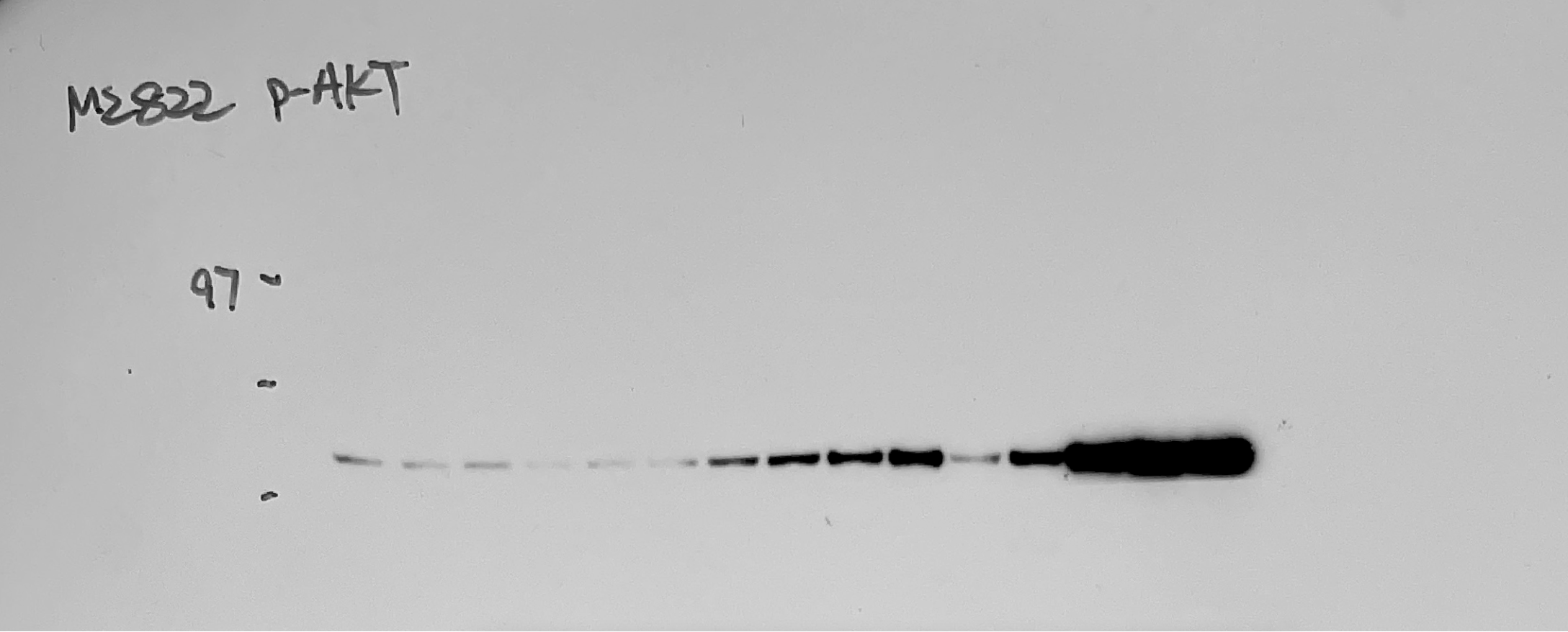

Supplement: Source data 1. [file elife-80014-data1.zip › Source data 1_western images/Figure 3C_pAKT.png]

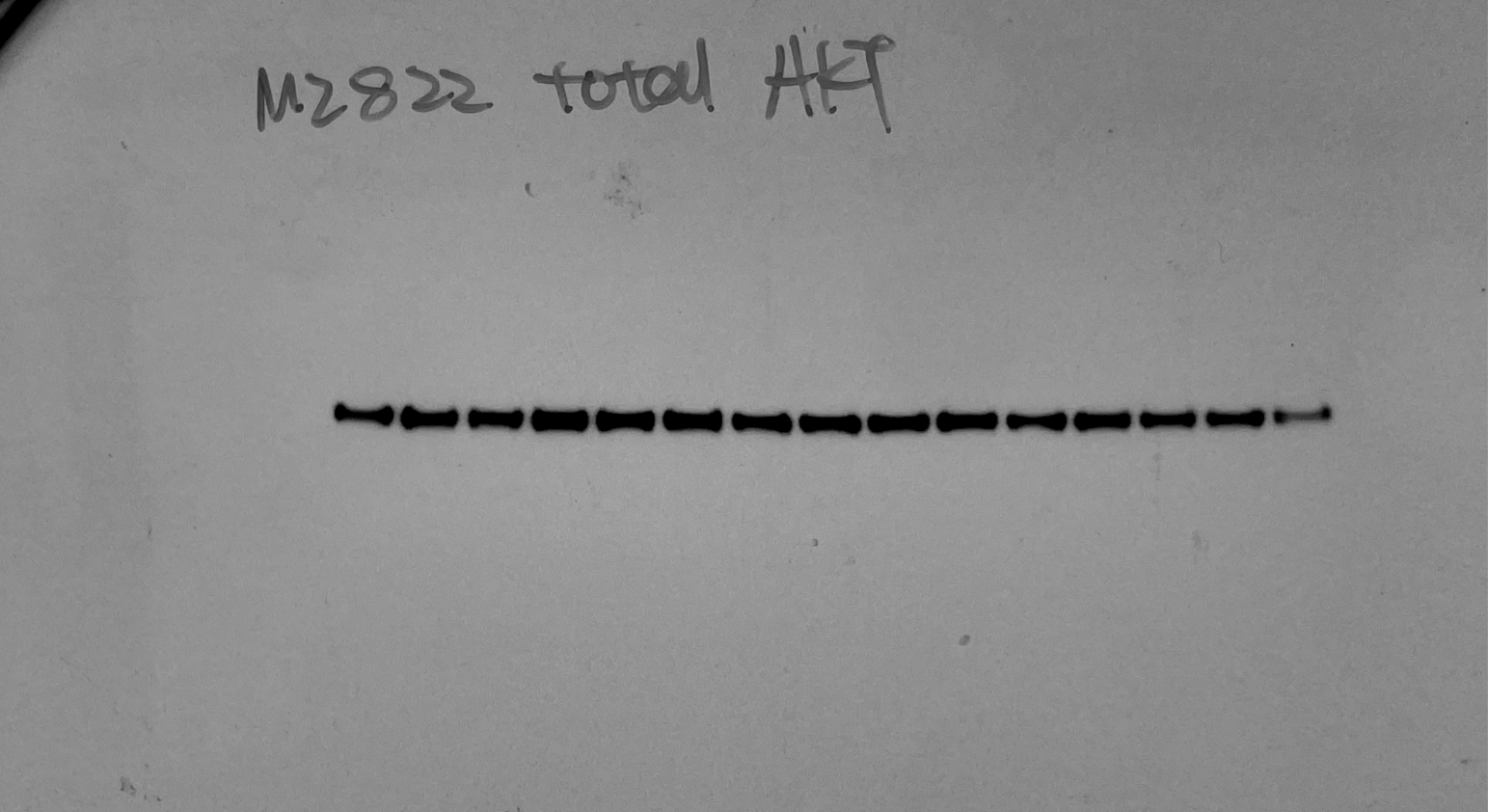

Supplement: Source data 1. [file elife-80014-data1.zip › Source data 1_western images/Figure 3C_AKT.png]

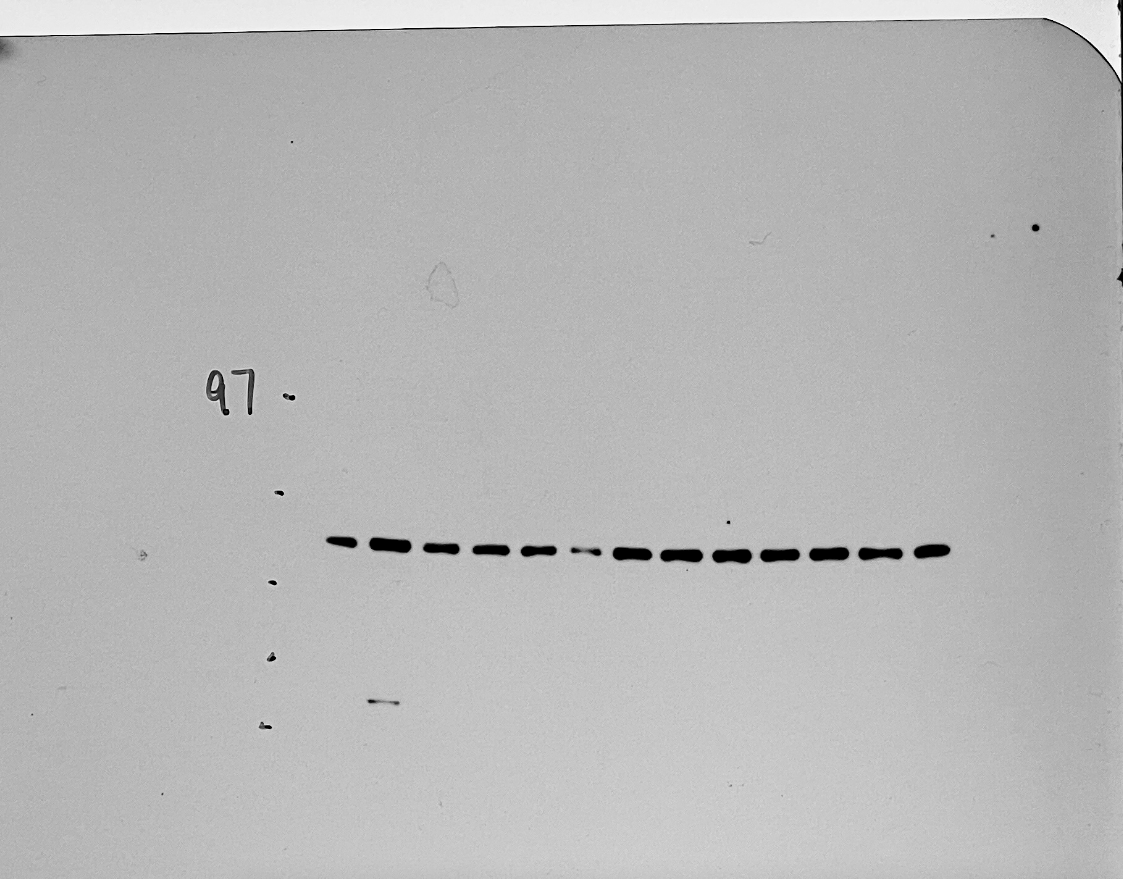

Supplement: Source data 1. [file elife-80014-data1.zip › Source data 1_western images/Figure 6G_AKT.png]

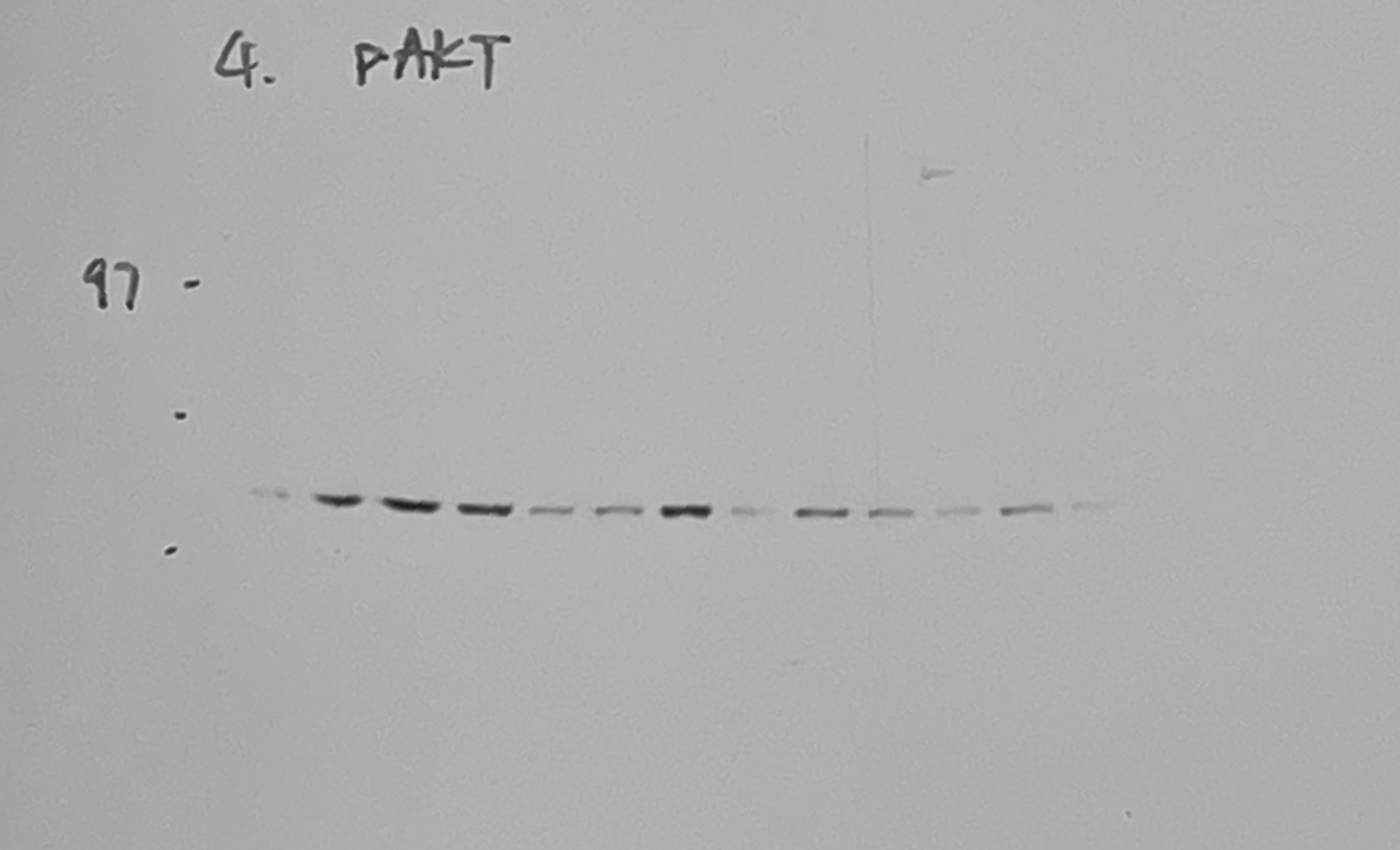

Supplement: Source data 1. [file elife-80014-data1.zip › Source data 1_western images/Figure 6G_pAKT.png]

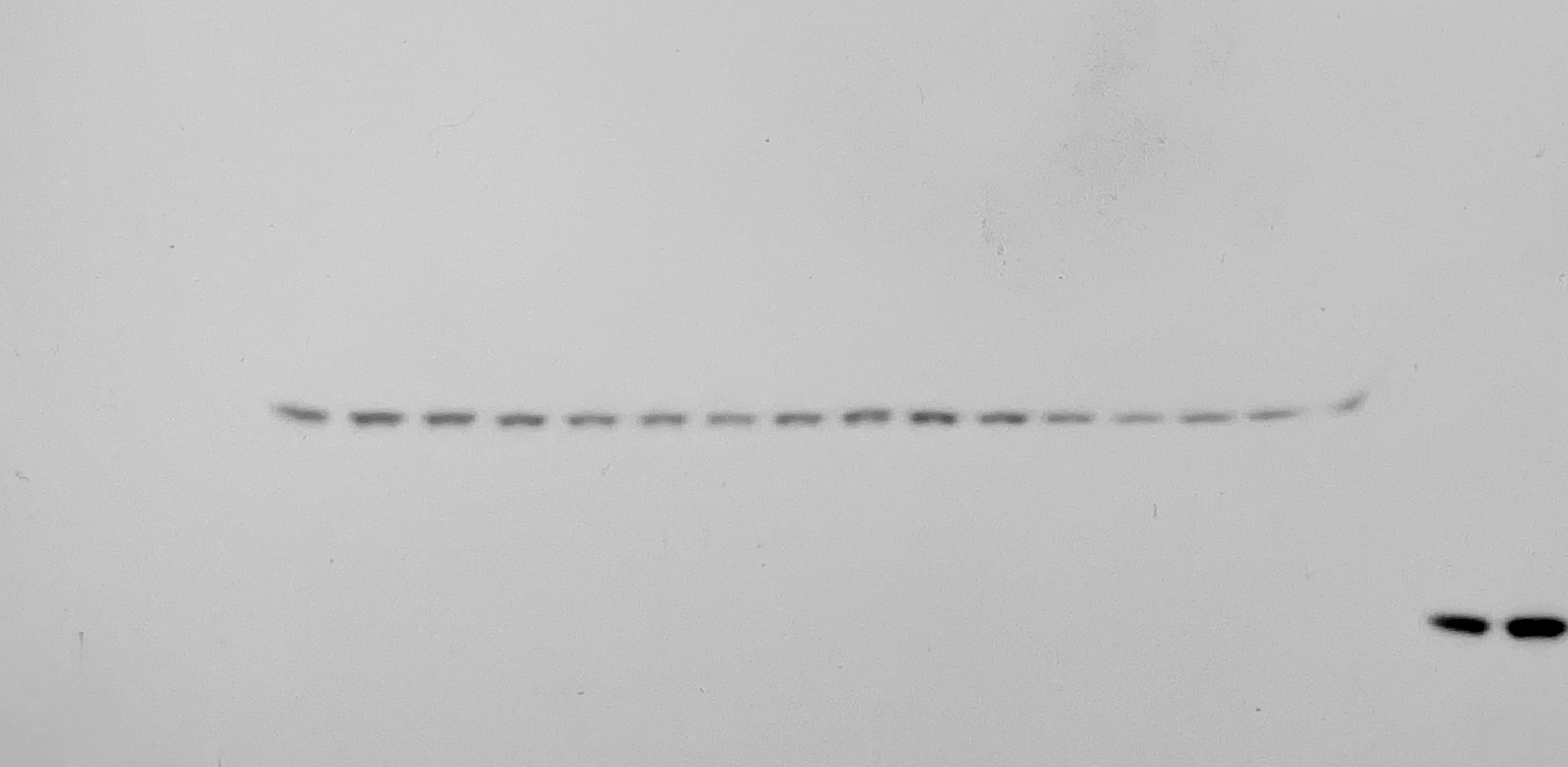

Supplement: Source data 1. [file elife-80014-data1.zip › Source data 1_western images/Figure 3A_S6.png]

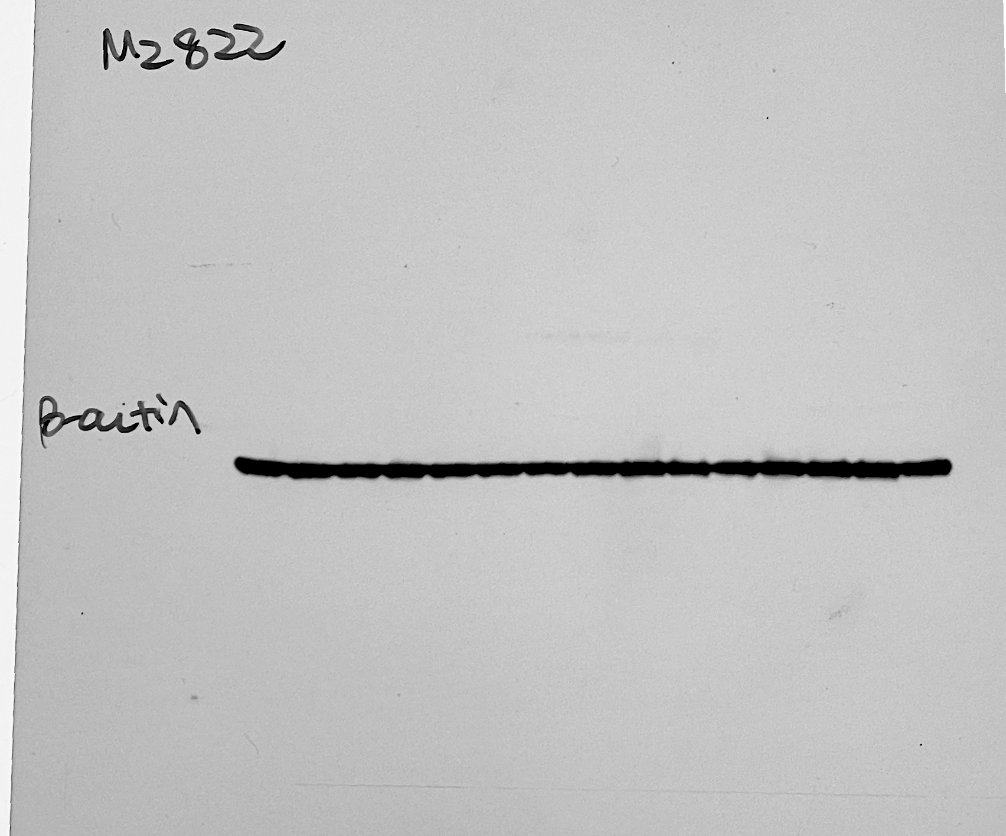

Supplement: Source data 1. [file elife-80014-data1.zip › Source data 1_western images/Figure 3C_actin.png]

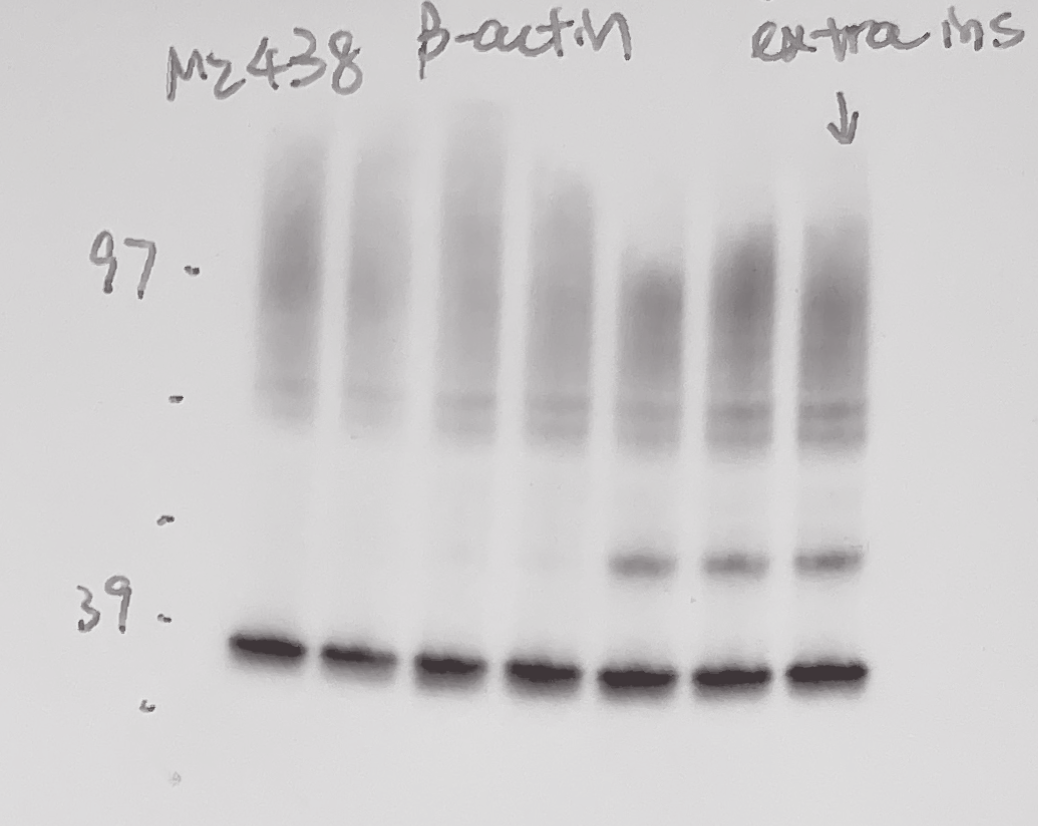

Supplement: Source data 1. [file elife-80014-data1.zip › Source data 1_western images/Figure 1B_actin.png]

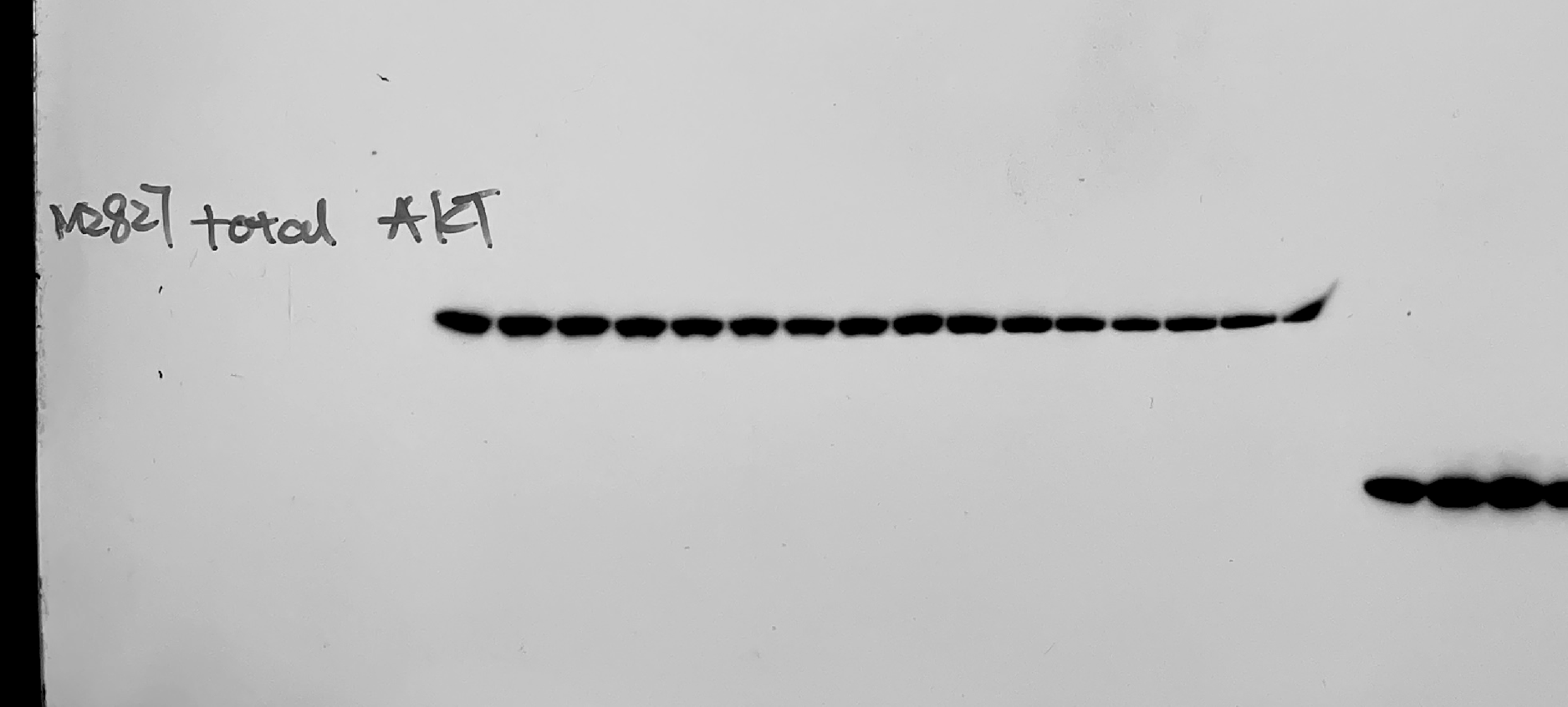

Supplement: Source data 1. [file elife-80014-data1.zip › Source data 1_western images/Figure 3A_AKT.png]

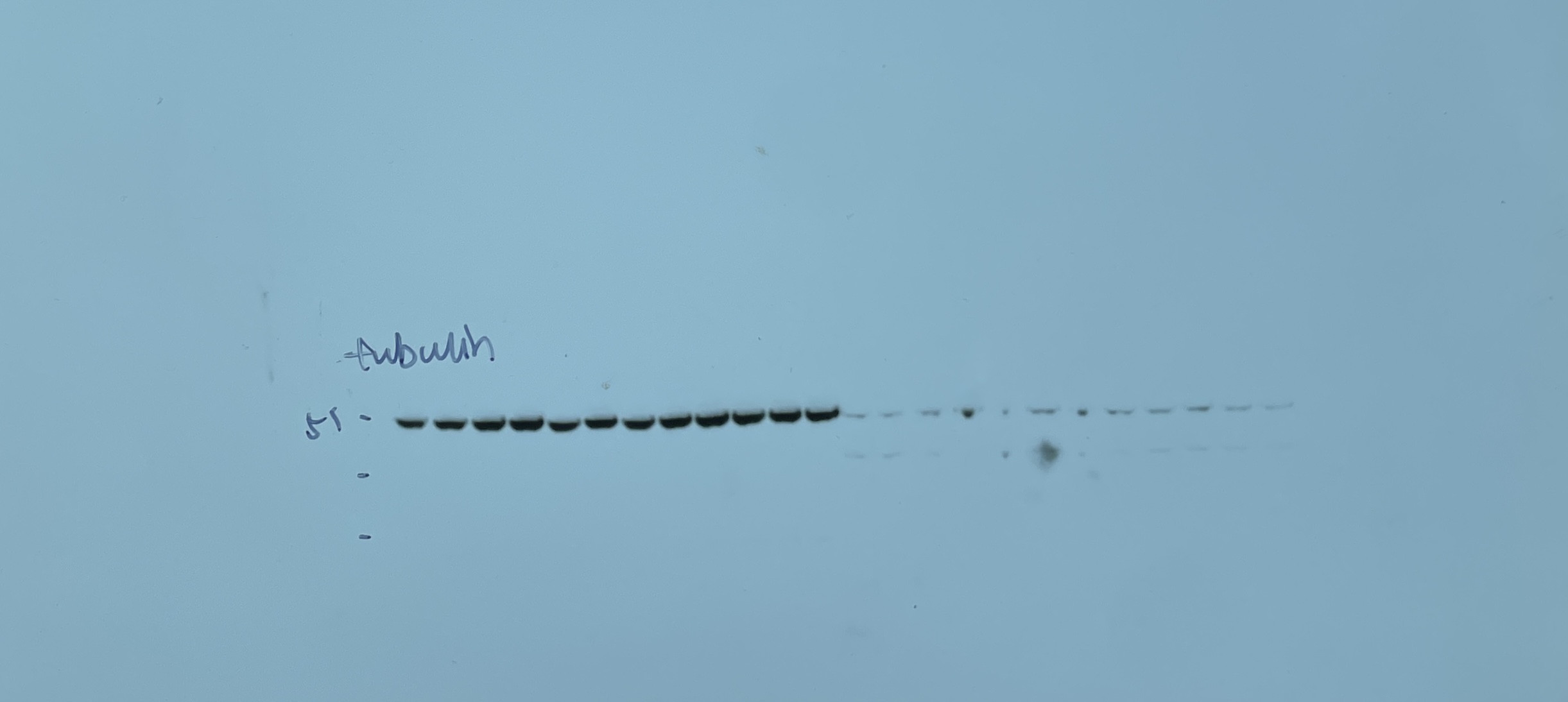

Supplement: Source data 1. [file elife-80014-data1.zip › Source data 1_western images/Figure 6F_Tubulin_revision_Sep2.jpg]
