# Supplementary material for: Phosphoproteomic mapping reveals distinct signaling actions and activation of muscle protein synthesis by Isthmin-1
Source: eLife. 2022 Sep 28;11:e80014. doi: 10.7554/eLife.80014 (PMC9592085; doi:10.7554/eLife.80014)
Supplement: Figure 6—source data 2. [file elife-80014-fig6-data2.pdf]

Fig 6F

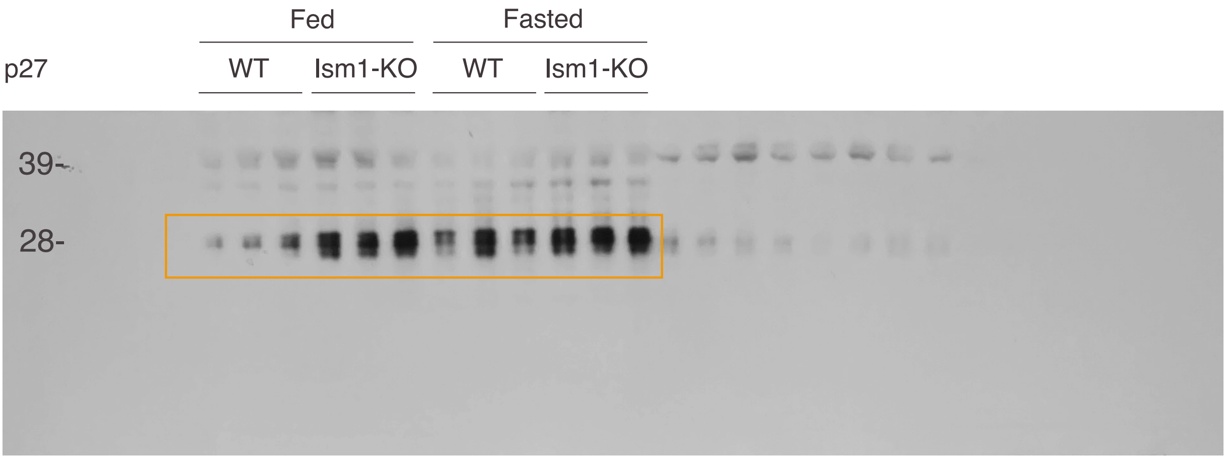

Tubulin

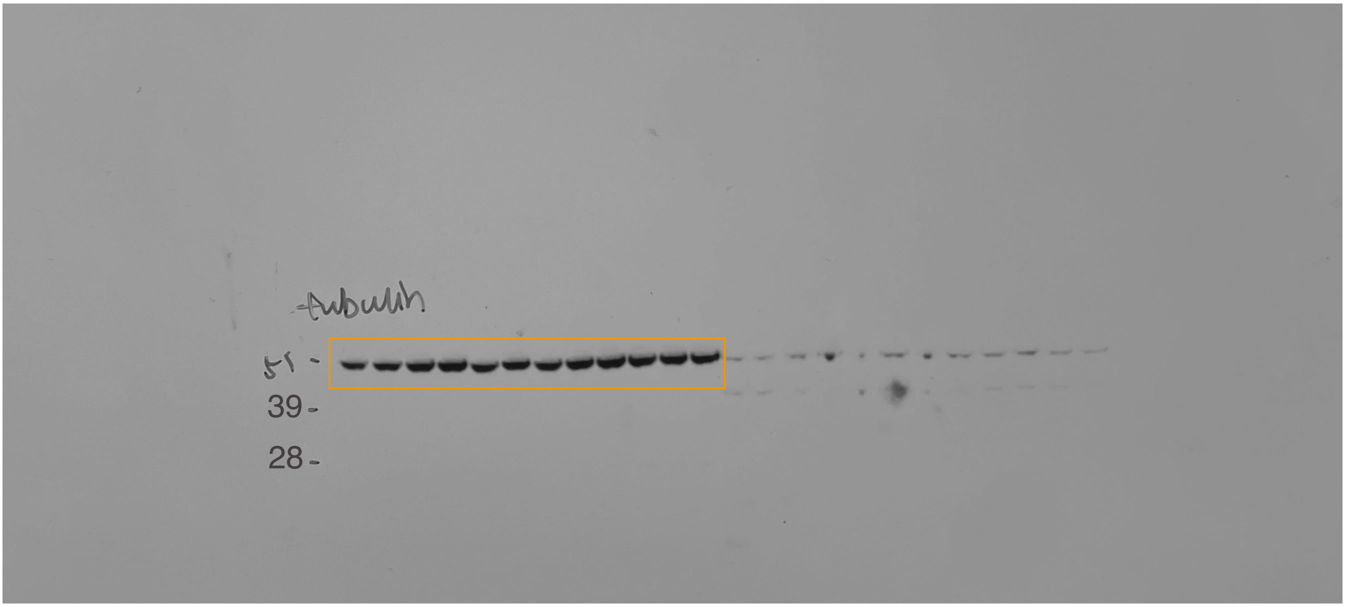

Fig 6G

p-AKT^{S473}

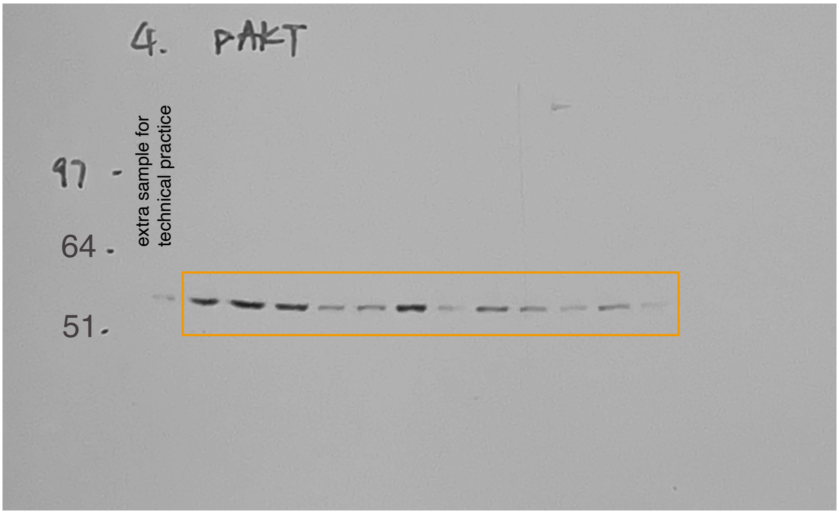

AKT

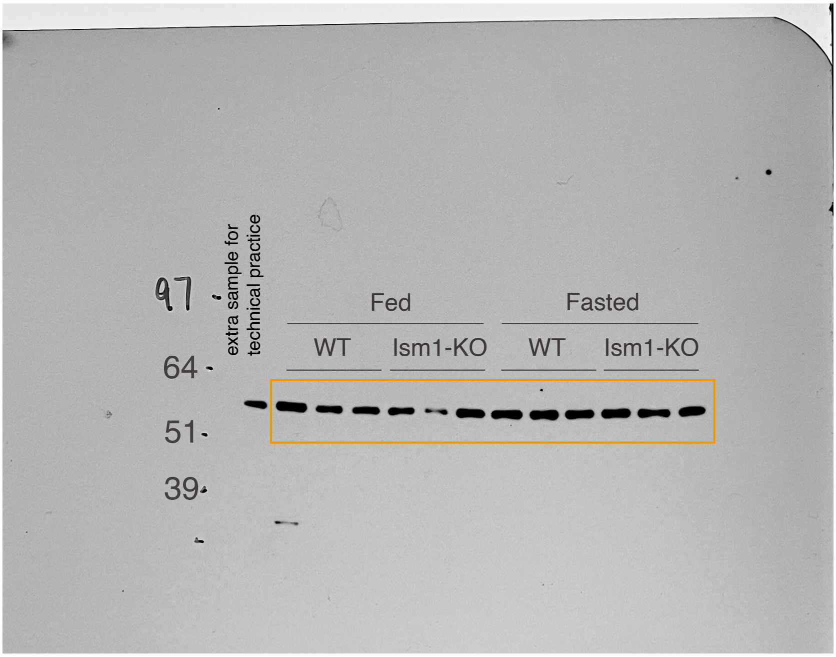

p-mTOR^{S2448}

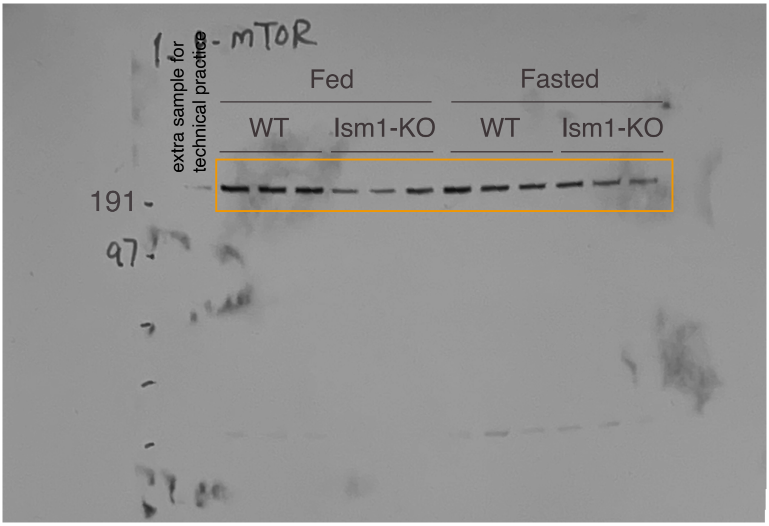

mTOR

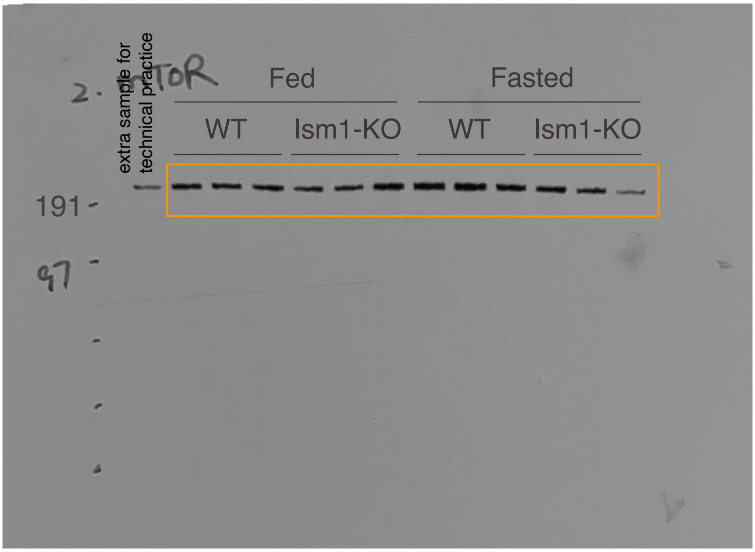

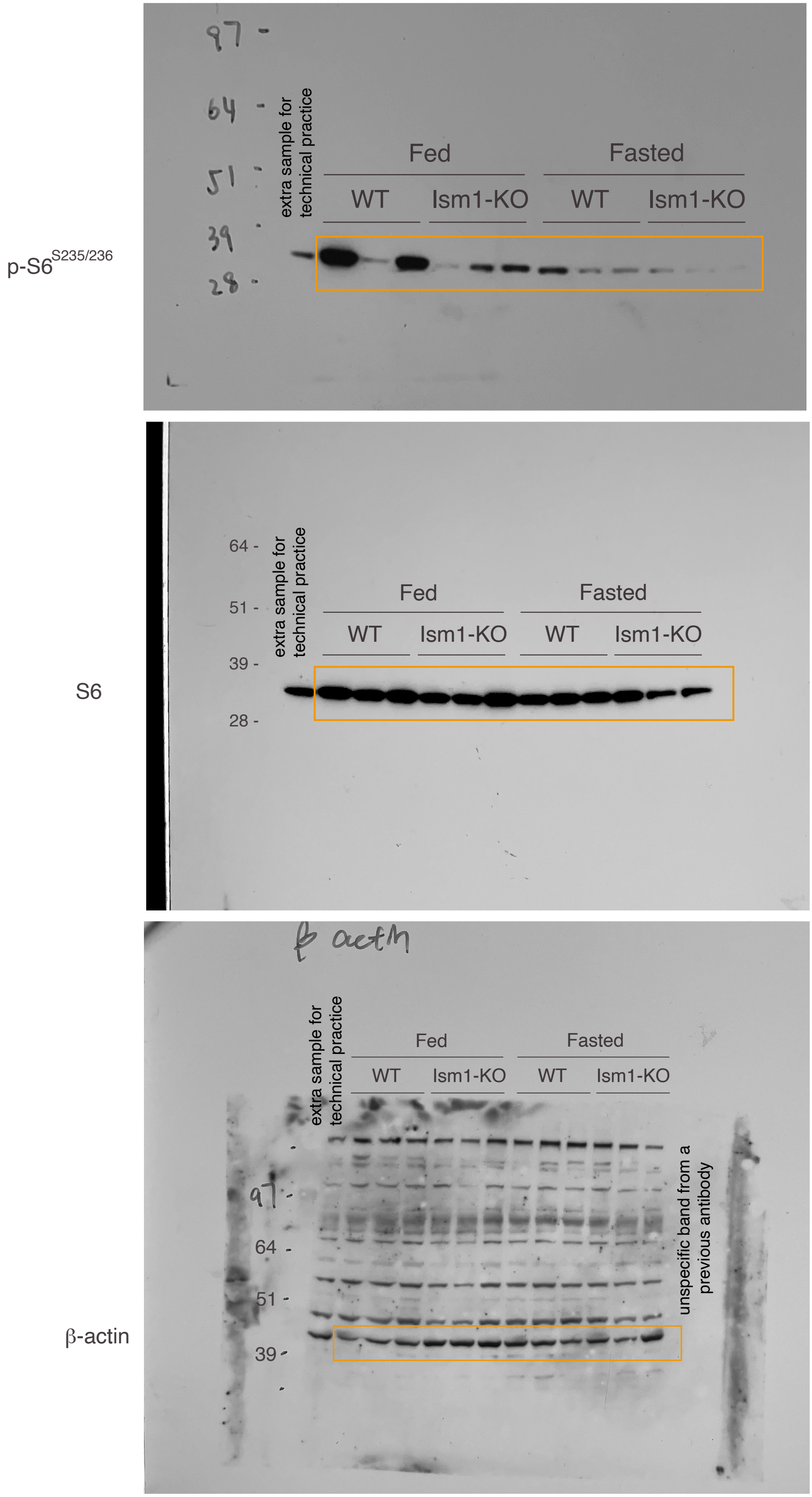

p-S6S235/236
97
64
51
39
28
extra sample for technical practice
Fed
Fasted
WT
Ism1-KO
WT
Ism1-KO
S6
64 -
51 -
39 -
28 -
extra sample for technical practice
Fed
Fasted
WT
Ism1-KO
WT
Ism1-KO
β actin
β-actin
extra sample for technical practice
Fed
Fasted
WT
Ism1-KO
WT
Ism1-KO
97
64 -
51 -
39 -
unspecific band from a previous antibody
