# Supplementary material for: Phosphoproteomic mapping reveals distinct signaling actions and activation of muscle protein synthesis by Isthmin-1
Source: eLife. 2022 Sep 28;11:e80014. doi: 10.7554/eLife.80014 (PMC9592085; doi:10.7554/eLife.80014)

qq-plots of normalized data

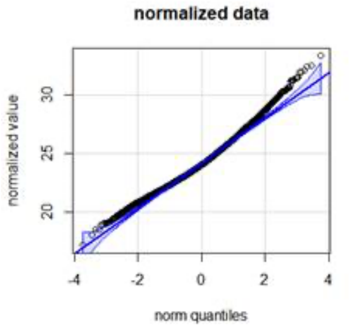

qq-plots of normalized and log2 transformed data

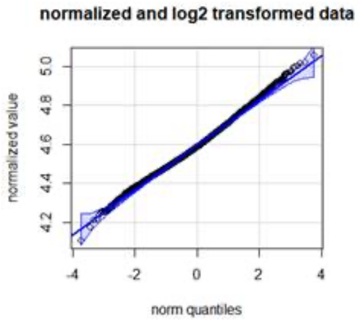

Boxplot of all phosphopeptides

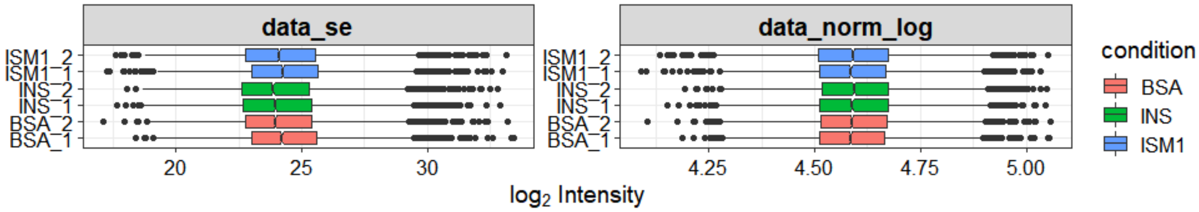

Figure A:

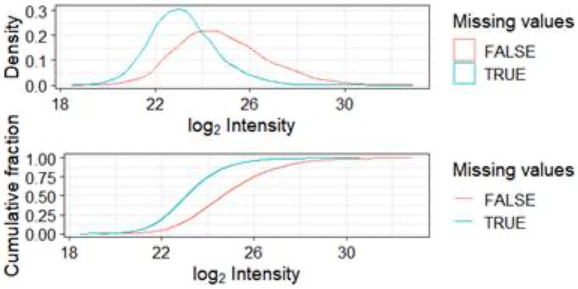

Figure B:

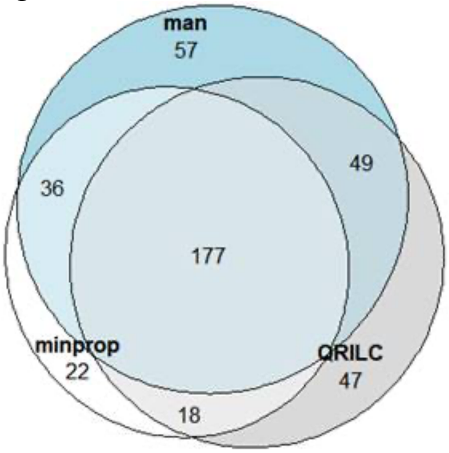

Supplement: Source data 2. [file elife-80014-data2.zip › Source data 2/Source data 2 figures .pdf]
